# Hip Muscle Strength Ratios Predicting Groin Injury in Male Soccer Players Using Machine Learning and Multivariate Analysis—A Prospective Cohort Study

**DOI:** 10.3390/muscles3030026

**Published:** 2024-09-02

**Authors:** Afxentios Kekelekis, Rabiu Muazu Musa, Pantelis T. Nikolaidis, Filipe Manuel Clemente, Eleftherios Kellis

**Affiliations:** 1 Laboratory of Neuromechanics, School of Physical Education and Sport Science at Serres, Aristotle University of Thessaloniki, 62100 Serres, Greece; ekellis@phed-sr.auth.gr; 2 Sport Injury Clinic for Prevention & Rehabilitation, 72100 Aghios Nikolaos, Greece; 3 Centre for Fundamental and Continuing Education, Universiti Malaysia Terengganu, Kuala Nerus 21030, Malaysia; rabiumuazu86@gmail.com; 4 School of Health and Caring Sciences, University of West Attica, 11521 Athens, Greece; pademil@hotmail.com; 5 Escola Superior Desporto e Lazer, Instituto Politécnico de Viana do Castelo, Rua Escola Industrial e Comercial d Nun’Álvares, 4900-347 Viana do Castelo, Portugal; filipe.clemente5@gmail.com; 6 Gdansk University of Physical Education and Sport, 80-336 Gdańsk, Poland; 7 Sport Physical Activity and Health Research & Innovation Center, 4900-347 Viana do Castelo, Portugal

**Keywords:** hip muscle strength ratios, machine learning, injury prediction, soccer, handheld dynamometer, groin injury

## Abstract

Despite ongoing efforts, the relationship between groin strength and injury remains unclear. The challenge of accurately predicting injuries presents an opportunity for researchers to develop prevention strategies to reduce the occurrence of such injuries. Consequently, this issue requires further investigation to obtain insights into effective mitigation strategies. In 120 male soccer players, the maximum isometric strength of the hip muscle groups was measured, and the strength ratios were calculated. Previous injury and anthropometric data were registered. Injury data were collected following the FIFA/UEFA consensus. *k*-nearest neighbor (*k*-NN) was used to predict the incidence of injury, while the significant predictive variables of the *k*-NN algorithm were fitted into a multivariate logistic regression model (LR) to analyze the likelihood of players sustaining a groin injury. The LR model determined two variables as significant predictors of groin injury. Players were less likely to sustain a groin injury by 76% for each decrease of the adductor/abductor isometric strength ratio in the non-dominant limb (OR = 0.238, CI 95% = [(0.098–0.572]). Players with a history of previous injury had a 67% greater risk of sustaining an injury (OR = 0.333, CI 95% = [(0.1068–1.038]). Isometric hip adductor and abductor strength imbalances of the non-dominant lower limb and a history of previous injury were risk factors for groin injury in soccer players.

## 1. Introduction

Hip and groin problems remain prevalent in team sports that require high-intensity tasks [1,2], including sprinting, sudden changes in direction [3], and kicking [4]. Groin injury incidence rates (IRs) range from 1.5 to 1.9 injuries per 1000 h of total exposure at elite [5] and amateur [6] levels, with a recurrence rate of 18% within two months [7]. These findings suggest the need to implement effective injury prevention programs to reduce the number and severity of groin injuries. To establish effective injury prevention strategies, it is essential that clinicians identify players at high risk of groin injury.

Deficits in muscle strength are commonly highlighted as significant risk factors for hip and groin injuries [1,8]. Strength-related variables may include the maximum absolute strength of the muscles involved [9], the strength difference between the two limbs [10,11], and the ratio of agonist to antagonist muscle strength [12]. Soccer players with low adductor strength encounter a 72% [9] or 80% [11] increased risk of sustaining a groin injury, although the magnitude of this association remains uncertain [13]. The link between hip/groin injury and variability in strength in both adductor and antagonist muscles is not thoroughly understood. Current evidence indicated that soccer players with hip abductor imbalances favoring the preferred kicking limb reported a 42% increased probability of sustaining a subsequent hip/groin injury [10]. Similarly, hockey players with weaker adductors relative to abductors were at a significantly increased risk of sustaining an adductor muscle strain [14]. Consequently, the association between strength and groin injury in soccer remains ambiguous, underscoring the importance of additional research in this domain.

Multiple factors may be accountable for the inconclusive results regarding the correlation between strength and hip/groin injury. Artificial intelligence (AI) and machine learning (ML) have been proposed to analyze problems where multiple risk factors and complexity are involved [15,16]. ML is an advanced tool for data analysis that utilizes algorithms that automatically learn from data to predict events [17]. ML algorithms have been recently applied to predict muscle injuries with high accuracy [18] or to identify players at risk for sustaining a hamstring injury [19]. Moreover, pre-season measurements demonstrated good to excellent accuracy in predicting acute or overuse injury amongst young elite soccer players [20] or whether a previously reported injury is likely to occur in the next season amongst professional NHL players [21]. Contrary to the previous promising studies, researchers reported low predictive accuracy in injury prediction [22] in identifying players at high risk for hamstring strain [23] or ACL injury [24]. To the best of our knowledge, the application of ML algorithms to describe the complex relationship between strength-related variables and hip and groin injuries has not been previously reported.

Muscle strength improvement is a vital component of pre-season and in-season exercise programs for enhancing performance as well as preventing injuries [25]. Nevertheless, considering the clinical perspective, screening for the player’s injury risk profile would guide practitioners in implementing successful preventive interventions. Comprehending this necessitates understanding the maximum strength capacity of the injured muscle, the relative absolute strength of each muscle group, and the balance between the agonist and the antagonist muscles. The primary objective of this study was to investigate the relationship between various factors and the incidence of groin injury in soccer players using ML algorithms. The use of advanced statistical algorithms, such as ML, may provide new insights into the identification of contributors to groin injuries. We hypothesized that players with deficits in maximum strength and strength imbalances between hip adductors and hip abductors were more likely to sustain a groin injury.

## 2. Methods

### 2.1. Study Design and Participants

**Figure 1:**
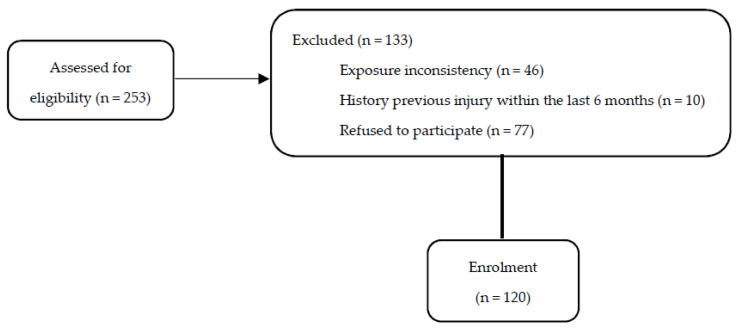
The consort flow diagram.

This prospective study follows the recommendations of the STROBE (Strengthening the Reporting of Observational Studies in Epidemiology) guidelines [26]. Male amateur soccer players over 14 years of age who participated in the regional soccer league and were injury-free the previous six months before the initial start of the pre-season were eligible to participate. During the off-season period of 2018/19 (June to August), 253 male players from 11 teams participating in a regional amateur league were contacted. Of these, 176 players initially agreed to participate. A total of 120 amateur soccer players gave their informed consent, and they were enrolled in this study, all of whom fell under tier 2 according to the Participants Classification Framework [27]. The consort flow diagram (Figure 1) describes the inclusion/exclusion procedures in detail. The protocol was approved by the ethics committee of Aristotle University in agreement with the Declaration of Helsinki.

### 2.2. Data Collection and Injury Data Registration

Anthropometric characteristics (age, stature, and body mass) were collected, and body mass index (BMI) was calculated. Players’ preferred leg(s), years of participation, and previous medical history were recorded. All participants performed four bilateral isometric strength tests at the start of the pre-season: hip adduction [28] (ADD), hip abduction [29] (ABD), hip flexion [30] (HFL), and knee flexion [31] (HMS) in a lengthening position. Muscle strength was measured using a KFORCE Muscle Controller (K-force, K-Invent Biomecanique, Montpellier, France). This is a hand-held dynamometer (HDD), which has shown high intra-rated (>0.79) and inter-rated (>0.72) reliability [32] and validity (ICC  >  0.79 and 0.89) for force and torque, respectively [33].

### 2.3. Testing Protocol

The maximum isometric strength tests of the hip muscles were conducted in this predetermined sequence. Isometric hip adduction (ADD) strength was evaluated from the supine position. The hips were slightly abducted to fit the angle of the tester’s elbows, as previously described by Nielsen and colleagues [28]. The isometric ABD strength was tested in the side-lying position, as previously described by Thorborg and colleagues [29]. The isometric HMS strength was tested in the prone position with the knee flexed 15° (0° = full knee extension), as previously described by Reurink and colleagues [31]. Isometric HFL strength was also evaluated from the supine position with hip and knee at 90°, as described by Thorborg and colleagues [30]. During all tests, the players were told to stabilize themselves by holding on to the sides of the table. In each testing position, the investigator’s hand was placed against the HHD to ensure correct action. The tester applied resistance in a fixed position, and the person being tested exerted a 3 s isometric maximum voluntary contraction (MVC) against the dynamometer and the tester. Participants were asked to resist the applied force (break test) [34]. Each test was performed bilaterally, starting with the right limb, and it was administered 2 times, separated by a 30 s resting period. A 2 min rest period between each of the four tests was applied to avoid a decrease in strength in the testing procedures due to potential fatigue [35]. The highest of the 2 valid MVCs was used for subsequent data analysis and treatment.

### 2.4. Injury Data Registration

Detailed standardized assessment instructions and specific documentation were provided to the medical teams of the clubs. A hip/groin injury was defined and classified in accordance with the Doha agreement meeting on groin pain in athletes [36]. During the competitive season, all injuries were diagnosed and confirmed by the medical staff of the football club or the medical staff of the local hospital following the recommendations adopted by the Doha agreement [36]. Injury data were collected every week, then verified and subsequently collected by the first author (A.K.), who visited each club weekly. Injury definition and assessment characteristics can be found in Supplementary Material S1a. All injuries were registered following the consensus statement on the definitions of injuries and data collection procedures adopted by FIFA [37]. The time spent by each player in training and match play was recorded by the club staff and was then verified by the first author on a weekly basis. The data collection form can be found in Supplementary Material S1b.

### 2.5. Statistical Analysis

Age, history of previous injury, physical characteristics (BMI), and the maximum isometric strength and strength ratios (Supplementary Material S1c) were the input variables, while groin injury status (injured, not injured) was the dependent variable in the model.

### 2.6. Development of the k-NN Model

The *k*-nearest neighbor (*k*-NN) algorithm is a fundamental supervised ML algorithm that can handle both regression and classification problems [38]. It is often referred to as ‘lazy learning’ or ‘instance-based learning’ due to its lack of a learning process. Instead, it stores the training dataset and performs computations at runtime [38,39]. The *k*-NN algorithm has demonstrated its effectiveness in mitigating non-linear relationships that commonly exist in datasets, making it a valuable tool for various classification problems across different domains [38,39]. In the *k*-NN algorithm, a sample is assigned to the most common class among its *k*-nearest neighbors in the data space. To determine these neighbors, a distance matrix is used to calculate and sort the distances of each sample from the others. For the development of the *k*-NN model, we utilized the Pycaret libraries via the Spyder IDE. Other statistical analyses were conducted using the XL STAT add-in software version 2014 for Windows. We selected *k* = 4 and used the Euclidean distance metric, which was determined to be the best fit for the model. To avoid overfitting, a five-fold cross-validation technique was employed, dividing the dataset into five distinctive folds and testing each one [40].

The data were divided into a 75:25 ratio for training and testing sets [41]. Specifically, we trained the model with 90 data points and tested it with 30 to predict the occurrence of groin injury. Furthermore, a sensitivity analysis was conducted using feature importance plots to evaluate the significance of isometric strength-related variables in influencing the model’s accuracy. These strength-related variables were then used as inputs to a multivariable binary logistic regression to predict the probability of players sustaining a groin injury.

### 2.7. Model Evaluation

To validate the *k*-NN model prediction, the following performance measures were calculated: the classification accuracy (ACC), area under the curve (AUC), recall, precision (PREC), and F1 score. ACC is the fraction of correctly classified instances. AUC is a curve that shows the model’s ability to separate classes. A recall is the proportion of true positives among actual positives, while PREC is the proportion of true positives among predicted positives. The F1 score is the harmonic mean of PREC and recall, and it measures the average accuracy of both classes.

## 3. Results

### 3.1. Descriptive Characteristics

Of the total of 120 participants (mean age: 20.0 ± 6.96 years; BMI: 22.53 ± 2.28 kg/m^2^, Height: 1.77 ± 0.07 m, body mass: 70.66 ± 10.08 kg), 22 (18.33%) experienced 25 groin injuries. Two players sustained a reinjury. The mechanisms of injury are presented in Table 1.

The means and standard deviations for the isometric strength measurements and strength ratios are presented in Table 2.

**Figure 2:**
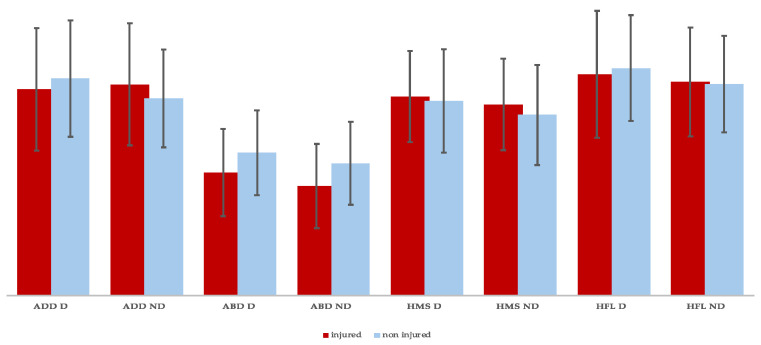
Between-groups absolute isometric strength differences (HFL = hip flexor; HMS = knee flexor; ABD = abductor; ADD = adductors; D = dominant; ND = non-dominant).

The injured players generated significantly lower MVCs in hip adduction and abduction of the dominant limb, the hip abduction of the non-dominant limb, and the hip flexors of the dominant limb (Figure 2).

**Figure 3:**
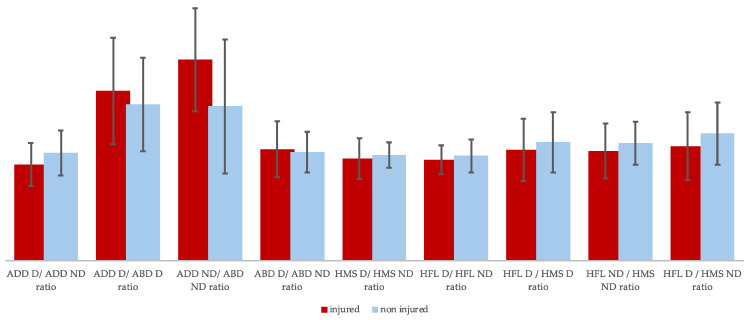
Between-groups strength ratio differences (HFL = hip flexor; HMS = knee flexor; ABD = abductor; ADD = adductors; D = dominant; ND = non-dominant).

Moreover, injured players demonstrate significant hip adduction/abduction imbalances between their dominant and non-dominant limbs (Figure 3).

### 3.2. The k-NN Model

The performance of the *k*-NN model in predicting the players’ chances of sustaining a groin injury is summarized in Table 3. The predictive model achieved a mean accuracy score of 55% and an area under the curve (AUC) of 0.43, indicating a reasonable injury prediction. The precision and recall scores indicated that the model predicted more than 60% of positive cases and correctly identified 80% of the actual positive classes.

**Figure 4:**
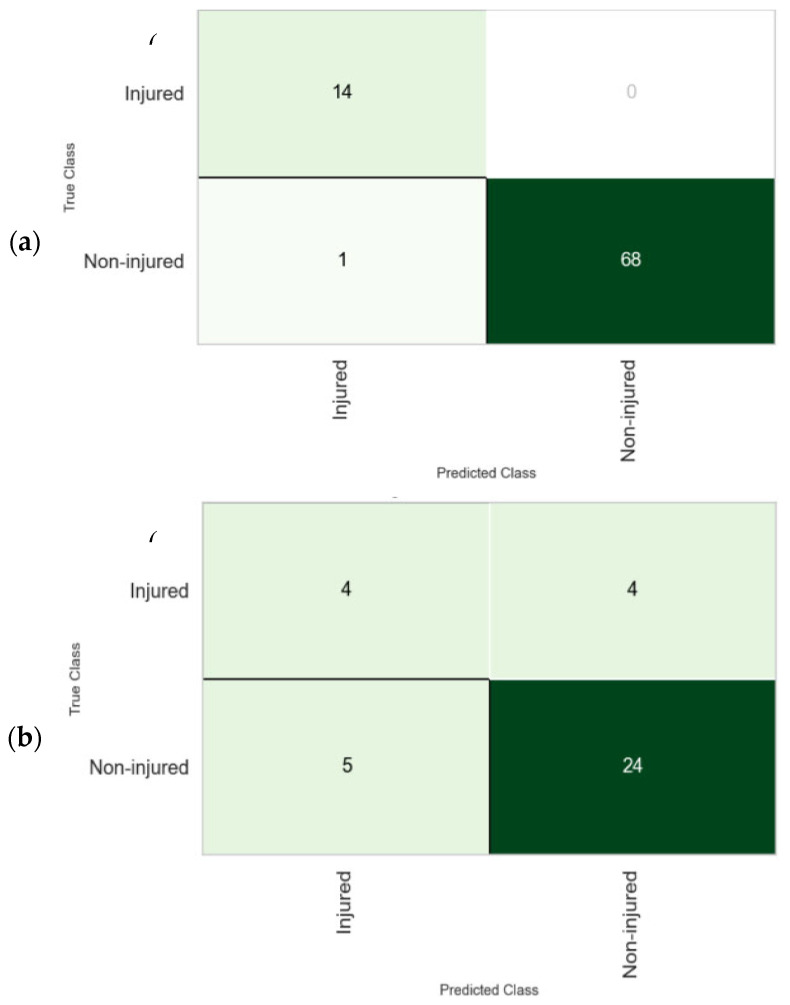
Confusion matrix of the *k*-NN: (**a**) training data set and (**b**) test data set.

The confusion matrix of the model developed after cross-validation is presented in Figure 4. This technique was employed to evaluate the performance of the classifier in predicting the groin injury of the players using the training and test data. The model correctly predicted 68 out of 74 non-injured players, indicating 1 misclassification. Fourteen (14) injured players were correctly classified, with no misclassification of the injured players during the training stage of the model. Similarly, the model correctly predicted 4 out of 8 injured players, whereas 5 non-injured players were misclassified out of 29. Overall, the model performed reasonably well in the classification task against the test data despite a relatively low number of observations, as well as the imbalance classes that existed within the data.

**Figure 5:**
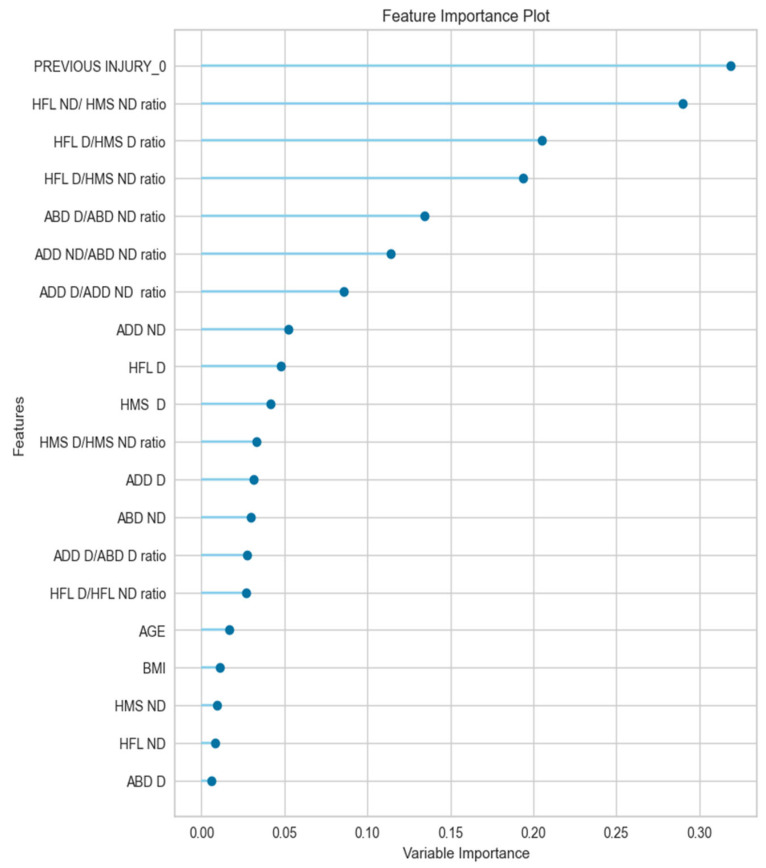
Variable contribution towards the RL model performance (HFL = hip flexor; HMS = knee flexor; ABD = abductor; ADD = adductor; D = dominant; ND = non-dominant).

Figure 5 demonstrates the graphical visualization of the variable’s contribution toward the performance of the model pipeline via the feature importance plot. It can be observed that 7 out of the 20 variables contributed more to the model performance (>8%) towards the probability of sustaining a groin injury. These seven variables were further analyzed using multivariate logistic regression analysis to determine their contribution to the probability of the players getting injured or not based on odds analysis.

### 3.3. The Regression Model

The results of the multivariate regression model are presented in Table 4. The results showed that players with a history of previous injury had a 67% increased risk of sustaining a groin injury (OR = 0.333, CI 95% = [(0.1068–1.038]). Additionally, players with a lower adductor/abductor isometric strength ratio in the non-dominant limb were less likely to sustain a groin injury by 76% (OR = 0.238, CI 95% = [(0.098–0.572]). No other significant contributor variables were found (*p* > 0.05). Overall, the model presented a well-fitting value (Hosmer–Lemeshow > 0.05), a good correct global classification (87%), and its discriminant capacity was also notable, with an AUC of 77% at a 95% confidence level. The model accounts for 22% of the players’ likelihood of sustaining groin injury or not, i.e., injured or non-injured (Negelkerke R^2^ = 22.00).

## 4. Discussion

The primary objective of this study was to examine the interrelationship amongst 20 variables through ML applications to predict groin injury in amateur soccer players. Our findings indicated that (a) the adductor/abductor isometric strength ratio of the non-dominant limb was a significant risk factor for groin injury; (b) soccer players with a history of groin injury were at a higher risk of sustaining a groin injury; (c) the isometric strength of either limb or the adductor/abductor ratio of the dominant limb were not significant injury risk factors.

### 4.1. Hip Muscle Strength Measurements and Ratios as Risk Factors for Groin Injury

The isometric strength of the hip adductors was not a significant contributor to the injury prediction model, which aligns with previous research findings [13,42]. Our results rebut previous findings that reported an increased risk of injury for players with a lower hip adductor muscle strength of the dominant limb [2,9,10,11,14], which may be attributed to the corresponding differences in methodology. In contrast to prior research, we included both agonist/antagonist strength ratios and absolute strength values, alongside hip flexor and knee flexor torque values and ratios, in our model (Figure 5). Previous research reported that those with lower abductor strength in their dominant/preferred limb relative to the other limb were at a higher risk of sustaining a groin injury [10], but other studies failed to confirm a similar association [11]. However, none of these studies examined strength imbalances in both limbs and their association with injury. A recent study observed a deficit in the hip adductor/abductor strength ratio during the middle and end of the season compared to the pre-season, which was more pronounced in the non-dominant limb [43]. This may assist in explaining the present findings, suggesting that strength levels may change during the competitive season, and these changes may differ not only between the hip adductors and abductors but also between the two limbs [43].

This study revealed that players with a lower adductor/abductor isometric strength ratio of the non-dominant limb had a 76% decreased risk of sustaining a groin injury (Table 4). The results of our study indicated that the balance ratio of the non-injured limb, typically the non-dominant limb, plays a crucial role in predicting groin injuries. Surprisingly, the predictive capacity was not determined by the absolute strength of the injured muscle or its antagonist but by the relative strength between the two antagonist muscle groups of the non-dominant site. Our findings indicate that strength imbalance between the hip adductors and abductors could impact the lumbo-pelvic movement, especially during demanding tasks such as acceleration [44], high-speed running [44], and change of direction (CoD) [44,45] (Table 1). Following the same line of thought, our study analyzed the correlation between the hip and knee flexors and their impact on lumbo-pelvic movement. Interestingly, while the regression analysis did not demonstrate significant predictive value for the ratios between the hip and knee flexors in terms of injury (Table 4), the machine learning algorithm indicated that the knee flexor/hip flexor strength ratio significantly contributed to the model’s performance (Figure 5). This suggests that alterations in muscle coordination and strength in the hip and pelvic area may contribute to increased forces in the adductor muscle–tendon units, leading to injury. However, it is apparent that further investigation is required to validate this suggestion.

### 4.2. Groin Injury Mechanism

Consistent with previous research [4], the leading injury mechanisms were changes in direction (CoD) and acceleration (Table 1). These tasks are characterized by high loads of the adductor longus and gracilis, as well as the encompassing passive structures of the groin area [44]. Furthermore, sprint accelerations show kinematics, kinetics, and adductor muscle forces that are like those observed during changes in direction maneuvers, implying that the phase of acceleration phase at the end of the change of direction movement might be responsible for the development of groin injury [44]. Recent studies have found two main mechanisms responsible for the development of groin pain: (1) high amounts of movement with eccentric contractions [46] and (2) rapid transitions between flexion and extension [47]. Both mechanisms are present during changes in direction and side kicking (passing of the ball), which occur repeatedly during training sessions or games [45,46,47]. Consequently, accumulative high muscle stress during eccentric adductor contractions during these accelerations results in high loads and increases the risk of groin injury [48]. The impact of the non-dominant limb in highly demanding soccer tasks, such as acceleration [44], CoD [45], and kicking [46,49], has been previously documented. During these movements, the non-dominant limb should support the body and stabilize the pelvis through closed kinetic chains. For example, during the first ground contact of sprint acceleration, the largest hip adductors’ forces were observed when there was a fast transition from hip abduction to adduction with the hip in extension [45]. Similarly, in cutting maneuvers and inside passing, the largest muscle activity of the adductors was found during rapid muscle lengthening [45]. Speculatively, a lower adductor strength of the non-dominant limb indicates a lower capacity of these muscles to withstand high forces when players change direction, especially when the muscles experience a large stretch while stabilizing the hip of the non-dominant limb during the last phase of the change in direction. Further research is necessary to explain the relationship between non-dominant lower limbs and the development of groin injuries.

### 4.3. The Value of Machine Learning in Injury Prediction

The present study utilized an ML algorithm to explore potential factors associated with groin injury, in contrast to earlier studies that relied on logistic regression [50,51]. The results of statistical algorithms rely on the number of input variables, their interactions, and their relationship with the occurrence of the injury. Hence, the results of this study were not directly comparable to those reported by previous studies. Interestingly, despite our assumptions, the inclusion of the absolute strength of the adductors or abductors in the model, along with the relative strength and other risk factors, did not emerge as a significant predictor of groin injuries (Figure 5). Consequently, there is doubt regarding whether players with lower absolute strength values were at a greater risk of groin injury.

ML algorithms have the advantage that they can model highly non-linear relationships, while logistic regression emphasizes inference [52]. The *k*-NN algorithm, which was implemented in this study, represents a novel approach to predicting groin injuries in soccer, enforcing previous efforts to predict injuries in professional adults [19] or junior players [20]. However, comparison between various studies is difficult due to differences in algorithm method, injury type, or level of play of the study sample. ML analyzes the significant variables and identifies those with a high predictive impact on the outcome. The algorithm considers both linear and non-linear relationships between the datasets during these analyses. In contrast, the multivariate logistic regression approach is employed as an odds analysis, predicting the likelihood of injury occurrence. However, it is important to note that the LR algorithm has a limitation: it can only extract data with a linear relationship. Consequently, when the relationship between variables is non-linear, the LR may not be able to identify its importance. Therefore, it is imperative to carefully consider the type of relationship between variables when choosing the appropriate statistical approach for injury prediction. Initially, we attempted to use all variables to fit the LR model, but the performance was subpar. Surprisingly, only one variable, namely “Previous injury”, was found to be significant. This finding emphasizes the limitations of the LR model in accurately capturing non-linear data patterns. However, we have successfully employed the LR model for the likelihood analysis, as demonstrated in the odds analysis.

### 4.4. Strengths and Limitations

A notable aspect of our study is the integration of the ratios of isometric strength variables into the predictive model (Table 4). This approach allows for a comprehensive analysis of all-encompassing variables pertinent to injury prediction. It is important to note that removing these added ratios may yield different results, as our analysis captures the intricate interrelationships among variables. Specifically, when a particular variable is deemed essential, other variables may be considered unimportant and vice versa. Researchers should take heed of these findings and consider the limitations of the LR model when dealing with non-linear data.

Several limitations have been encountered in our research. It is acknowledged that the study included a relatively small sample of players, resulting in a few injury incidents. It is important to note that this study investigated players who were part of an 11-team amateur league. Despite contacting all teams, we managed to recruit six teams, which accounts for a reasonable 54.5% participation rate. Another limitation is that, by defining injuries as time-loss injuries, we did not consider players’ problems that required medical assistance but did not result in time loss. Furthermore, it should be noted that the measurements were performed in a field setting, which precluded the application of any belt fixation, maintaining the reliability between the two methods [53]. The lack of complete medical history resulted in missing injury site details, preventing us from incorporating limb dominance as an independent variable in the ML algorithm. In upcoming research, researchers may analyze the accuracy of ML algorithms in predicting injury side. These limitations should be taken into consideration when interpreting our findings. Conversely, the study’s internal validity was a positive aspect because the same investigator conducted all the measurements.

A promising direction for future research on athletes’ injury risk profile could entail a detailed pre-season multivariate screening strategy that incorporates validated battery tests and advanced biomechanical assessment tools that replicate the mechanism of groin injury. Apparently, further investigation is required to validate our hypothesis, derived from our findings, that muscle strength imbalances over the hip and lumbo-pelvic complex may have an impact on increased forces in the adductor muscle–tendon unit, leading to injury. Obtaining insights into the potential contributors to groin injury may simply guide stakeholders in implementing effective prevention strategies.

## 5. Conclusions

This study utilized an ML algorithm to examine the contribution of 20 variables and indices of hip and knee strength to groin injury in amateur soccer players followed for one season. The low isometric strength ratio of adductor/abductor in the non-dominant limb and history of prior injuries were associated with injury, thus emphasizing the importance of addressing strength imbalances in the non-dominant limb. Injury mechanisms involving CoD and acceleration were predominant. Our findings showcase the potential of advanced ML techniques for accurate injury prediction, offering valuable insights for targeted prevention strategies and player well-being.

## Figures and Tables

**Table 1:** Mechanism of injury.

Mechanism of Groin Injury	N
Change of direction (CoD)	12
Acceleration	4
Stretching	3
Kicking	2
Inside pass	2
Decceleration	2
Total	25

**Table 2:** Mean (± standard deviation) values for the injured and non-injured groups for each dependent variable. (HFL = hip flexor; HMS = knee flexor; ABD = abductor; ADD = adductors; D = dominant; ND = non-dominant).

Variable	Mean-SD	
	Injured (*n* = 98)	Non-Injured (*n* = 22)
ADD D	25.40 ± 7.56	26.74 ± 7.16
ADD ND	26.02 ± 7.51	24.30 ± 6.01
ABD D	15.16 ± 5.39	17.62 ± 5.22
ABD ND	13.50 ± 5.20	16.29 ± 5.10
HMS D	24.51 ± 5.60	23.99 ± 6.38
HMS ND	23.55 ± 5.63	22.28 ± 6.16
HFL D	27.26 ± 7.79	28.02 ± 6.51
HFL ND	26.33 ± 6.67	26.05 ± 5.96
ADD D/ADD ND ratio	0.99 ± 0.23	1.11 ± 0.22
ADD D/ABD D ratio	1.75 ± 0.48	1.61 ± 0.55
ADD ND/ABD ND ratio	2.07 ± 0.69	1.59 ± 0.53
ABD D/ABD ND ratio	1.15 ± 0.21	1.12 ± 0.29
HMS D/HMS ND ratio	1.05 ± 0.13	1.09 ± 0.21
HFL D/HFL ND ratio	1.04 ±0.17	1.08 ± 0.15
HFL D/HMS D ratio	1.14 ± 0.31	1.22 ± 0.32
HFL D/HMS ND ratio	1.13 ± 0.22	1.21 ± 0.28
HFL ND/HMS ND ratio	1.18 ±0.32	1.31 ± 0.35

**Table 3:** Performance evaluation of the *k*-NN model for predicting adduction injury risk among players.

	Accuracy	AUC	Recall	Prec.	F1
Mean	0.556	0.425	0.609	0.806	0.688
Std	0.131	0.278	0.941	0.108	0.197

**Table 4:** The results of the multivariate regression model indicating whether each input variable is a significant contributor.

						95% Confidence Interval	
Variables	B	SE	Z	*p*	Odds Ratio	Lower	Upper
Intercept	2.5628	1.744	1.4697	0.142	12.972	0.4253	395.618
History	−1.0997	0.58	−1.8952	0.050 *	0.333	0.1068	1.038
HFL ND/HMS ND ratio	0.1479	1.703	0.0869	0.931	1.159	0.0412	32.626
HFL D/HMS D ratio	0.0499	1.354	0.0368	0.971	1.051	0.0739	14.943
HFL D/HMS ND ratio	1.1717	1.55	0.7558	0.45	3.228	0.1546	67.366
ABD D/ABD ND ratio	0.4482	1.113	0.4028	0.687	1.566	0.1768	13.862
ADD ND/ABD ND ratio	−1.4362	0.448	−3.2047	0.001 *	0.238	0.0988	0.572

Note. * *p* < 0.05; Nagelkerke R^2^ = 22.00; Hosmer–Lemeshow (*p* = 0.34); AC = 83%; AUC = 0.774. HFL = hip flexor; HMS = knee flexor; ABD = abductor; ADD = adductor; D = dominant; ND = non-dominant; B = beta coefficient; SE = standard error; Z = Z value; *p* = level of significance.

## Data Availability

The data presented in this study are available upon request from the corresponding author.

## Supplementary Materials

The following supporting information can be downloaded at: https://www.mdpi.com/article/10.3390/muscles3030026/s1, S1a for definitions and assessment; S1b injury report and S1c variables applied. All supplementary tables are included in a single file.
