# Supplementary material for: Hip Muscle Strength Ratios Predicting Groin Injury in Male Soccer Players Using Machine Learning and Multivariate Analysis—A Prospective Cohort Study
Source: Muscles. 2024 Sep 2;3(3):297–309. doi: 10.3390/muscles3030026 (PMC12225289; doi:10.3390/muscles3030026)
Supplement: Supplementary file 1 [file muscles-03-00026-s001.zip › muscles-3129702-supplementary.pdf]

Definitions (Weir A. et al., 2015)

Defined clinical entities for groin pain.

1. *Adductor-related groin pain*: Adductor tenderness and pain on resisted adduction testing.
2. *Iliopsoas-related groin pain*: Iliopsoas tenderness. The iliopsoas-related groin pain is more likely if there is pain on resisted hip flexion AND/OR pain on stretching the hip flexors.
3. *Inguinal-related groin pain*: Pain location in the inguinal canal region and tenderness of the inguinal canal. No palpable inguinal hernia is present. Inguinal-related groin pain is more likely to be identified if the pain is aggravated with resistance testing of the abdominal muscles or on Valsalva/cough/sneeze.
4. *Pubic-related groin pain*: Local tenderness of the pubic symphysis and the immediately adjacent bone.
5. *Hip–related groin pain* should always be considered as a possible cause of groin pain.

Assessment tool (Brukner PB, Kahn K. *Groin pain. Clinical sports medicine. 5th edition*. Sydney: McGrawl-Hill, 2017)

1. Observation: a) standing, b) walking, c) squatting, running, jumping.
2. Active movements: a) hip flexion/extension, b) hip adduction/abduction, c) hip internal/external rotation, d) lumbar spine movements.
3. Passive movements: a) passive movement-adductor muscle stretch, b) modified Thomas test.
4. Resisted movements: a) resisted movement single-leg, b) resisted movement-squeeze test (with hips in neutral and 45^0 flexion, c) hip flexion, d) abdominal muscles.
5. Palpation: a) adductors, b) psoas above inguinal ligament, c) psoas below inguinal ligament, d) inguinal region -external inguinal ring, e) inguinal region – scrotal investigation, f) pubic symphysis
6. Hip-related groin pain: a) hip joint assessment.
7. Screening for other pathology: a) fractures, stress- fractures, pubic ramus, b) thoracic spine, c) lumbar spine, d) Sacroiliac joint

Supplementary Material S1a. Definitions and Standardized Assessment tool

	player	Age	Position	Height	Weight	BMI	Dominant	Previous Injury *	Date of injury	injury	Mechanism of injury	Game or Training	Re-injury	RTP days	Use of imagine
1	Player 1	20	Central Midfielder	1.67	59	21.2	RIGHT	YES	22.08.18	1st grade R ARGI*	CoD*	T*	NO	5	no
2	Player 2	28	Defense Middle	1.71	73	25.0	RIGHT	YES	6.10.18	1st grade R ARGI	CoD	T	NO	3	no
3	Player 3	28	Attacking winger	1.74	71	23.5	RIGHT	YES	6.10.18	1st grade R ARGI	CoD	T	NO	6	no

	Player 3	28	Attacking winger	1.74	71	23.5	RIGHT	YES	13.10.18	2nd grade R ARGI	CoD	Game	YES	18	yes
4	Player 4	20	Attacking Winger	1.78	67	21.1	RIGHT	YES	24.10.18	1st grade R ARGI	CoD	T	NO	3	no
5	Player 5	17	Defence winger	1.78	78	24.6	RIGHT	YES	16.12.18	1st grade L ARGI	CoD	Game	NO	3	no
6	Player 6	16	Central defender	1.84	90	26.6	RIGHT	YES	10.02.19	1st grade R ARGI	acceleration	Game	NO	3	no
7	Player 7	14	attacking winger	1.70	60	20.8	LEFT	YES	10.02.19	1st grade R ARGI	CoD	Game	NO	7	no
8	Player 8	17	Central forwrad	1.83	71	21.2	RIGHT	YES	24.02.19	1st grade L ARGI	acceleration	Game	NO	3	no
9	Player 9	16	Central attacking	1.80	73	22.5	LEFT	YES	3.04.19	1st grade L ARGI	stretching	Game	NO	5	no
10	Player 10	15	Center Middfielder	1.72	70	23.7	RIGHT	NO	6.09.18	1st G R Iliopsoas RGI*	kicking	T	NO	6	no
	Player 10	15	Center Middfielder	1.72	70	23.7	RIGHT	NO	27.11.18	1st grade R ARGI	CoD	T	YES	9	yes
11	Player 11	21	Goalkeaper	1.91	89	24.4	RIGHT	YES	4.10.18	1st grade L ARGI	kicking	T	NO	3	no
12	Player 12	18	Defence winger	1.75	68	22.2	RIGHT	NO	14.10.18	1st grade L ARGI	CoD	Game	NO	5	no
	Player 12	18	Defence winger	1.75	68	22.2	RIGHT	NO	23.03.19	1st grade R ARGI	acceleration	Game	NO	3	no
13	Player 13	18	Attacking Middle center	1.85	72	21.0	RIGHT	YES	10.10.18	1st grade L inguinal RGI	stretching	Game	NO	7	no
14	Player 14	34	Central defender	1.75	62	20.2	RIGHT	YES	16.10.18	1st grade L ARGI	deceleration	T	NO	5	no
15	Player 15	23	Defence winger	1.75	65	21.2	RIGHT	YES	20.11.18	1st grade R ARGI	stretching	Game	NO	3	no
16	Player 16	17	Attacking Winger	1.72	55	19.0	RIGHT	NO	1.02.19	1st grade L ARGI	inside pass	T	NO	7	no
17	Player 17	28	Central attacking	1.84	82	24.2	RIGHT	YES	14.12.18	2nd grade L ARGI	CoD	Game	NO	19	yes
18	Player 18	15	Attacking Winger	1.80	65	20.1	RIGHT	NO	30.01.19	1st grade R ARGI	deceleration	Game	NO	7	no
19	Player 19	16	Attacking Winger	1.65	52	19.1	RIGHT	NO	8.11.18	1st grade R ARGI	CoD	T	NO	2	no

20	Player 20	15	Defence winger	1.72	63	21.3	LEFT	YES	20.01.19	1st grade L ARGI	acceleration	T	NO	3	no
21	Player 21	16	Center Middfielder	1.70	57	19.7	RIGHT	NO	14.02.18	1st grade L inguinal RGI	CoD	Game	NO	2	no
22	Player 22	22	Attacking Middle center	1.78	85	26.8	LEFT	NO	2.10.18	1st grade R Iliopsoas RGI	inside pasS	T	NO	6	no

All injuries that met the inclusion criteria were reported by team medical staff using an injury report form that included the date and time of injury (if during a game or training session), the date of return to training and match play, injury location (adductor related, iliopsoas related, inguinal related, pubic related, hip related, other), injury severity (grade 1-3), injury recurrence (yes/no), and use of imaging to confirm the diagnosis (yes/no).

Abbreviations: **BMI**: Body mass index, RGI: Related Groin Injury, AGRI: Adductor related Groin injury, CoD : Change of direction, T: training

Previous Injury *: Due to limited information provided during the players' initial history (interview) process, we were unable to confirm the injury site.

Supplementary Material S1b. Injury report

1	Previous hamstring Injury
2	Age
3	BMI
4	Adductors dominant limb (ADD-D),
5	Adductors non-dominant limb (ADD-ND),
6	Abductors dominant limb (ABD-D),
7	Abductors non-dominant limb (ABD-ND),
8	Hamstrings dominant limb (HMS-D),
9	Hamstrings non-dominant limb (HMS-ND),
10	Hip flexors dominant limb (HFL-D),
11	Hip flexors non-dominant limb (HFL-ND),
12	Adductors dominant limb/ Adductors non-dominant limb (ADD-D/ADD-ND) ratio,
13	Adductors dominant limb /abductors dominant limb ratio (ADD-D/ABD-D) ratio,
14	Adductors non-dominant limb /Abductors non dominant limb (ADD-ND/ABD ND) ratio,

15	Abductors dominant limb/ Abductors non-dominant limb (ABD-D/ABD ND) ratio,
16	Hip Flexors dominant limb/ Hip Flexors non-dominant limb (HFL-D/HFL-ND) ratio,
17	Hip Flexors dominant limb/ Hamstrings dominant limb (HFL-D/HMS-D) ratio,
18	Hip Flexors dominant limb/ Hamstrings non-dominant limb (HFL-D/HMS-ND) ratio,
19	Hip Flexors non-dominant limb/ Hamstrings non-dominant limb (HFL-ND/ HMS-ND) ratio,
20	Hamstrings dominant limb/ Hamstrings non-dominant limb (HMS-D/HMS-ND) ratio,

Supplementary Material S1c. Input Variables applied to predict hamstring injury risk.
